# Clinical Significance of the Prealbumin Level in Gastric Cancer Patients Who Receive Curative Treatment

**DOI:** 10.1007/s12029-021-00777-w

**Published:** 2021-12-18

**Authors:** Toru Aoyama, Masato Nakazono, Kenki Segami, Shinsuke Nagasawa, Kazuki Kano, Kentaro Hara, Yukio Maezawa, Itaru Hashimoto, Hideaki Suematsu, Hayato Watanabe, Keisuke Komori, Hiroshi Tamagawa, Norio Yukawa, Yasushi Rino, Takashi Ogata, Takashi Oshima

**Affiliations:** 1 Department of Surgery, Yokohama City University, Yokohama, Japan; 2 Department of Gastrointestinal Surgery, Kanagawa Cancer Center, Yokohama, Japan

**Keywords:** Astric cancer, Prealbumin, Survival

## Abstract

**Background:**

We investigated the clinical influence of the prealbumin level on the gastric cancer survival and recurrence after curative treatment.

**Methods:**

This study included 447 patients who underwent curative treatment for gastric cancer between 2013 and 2017. The risk factors for the overall survival (OS) and recurrence-free survival (RFS) were identified.

**Results:**

A prealbumin level of 20 mg/dl was regarded as the optimal point of classification, considering the 3- and 5-year survival rates. The OS rates at 3 and 5 years after surgery were 80.7% and 65.0% in the low-prealbumin group, respectively, and 93.1% and 87.9% in the high-prealbumin group, respectively, a statistically significant difference (*p* < 0.001). The RFS rates at 3 and 5 years after surgery were 71.7% and 68.0% in the low-prealbumin group, respectively, and 90.1% and 84.7% in the high-prealbumin group, respectively, a statistically significant difference (*p* = 0.031). A multivariate analysis demonstrated that the prealbumin level was a significant independent risk factor for the OS and RFS. In addition, the rate of introduction of adjuvant chemotherapy was significantly lower and the frequency of peritoneal recurrence and lymph node recurrence significantly higher in the low-prealbumin group than in the high-prealbumin group.

**Conclusion:**

Prealbumin is a risk factor for the survival in patients who undergo curative treatment for gastric cancer. It is necessary to develop an effective plan of perioperative care and surgical strategy according to the prealbumin level.

## Introduction

Gastric cancer is the third-most common cancer and the second leading cause of cancer-related death in the world. Every year, 1 million new cases of gastric cancer occur, with 800,000 gastric cancer deaths noted worldwide [1, 2]. Gastrectomy with D2 lymphadenectomy with or without pre- and/or postoperative adjuvant treatment is a global standard treatment for resectable gastric cancer. However, even when patients receive curative treatment, more than half develop recurrence [3–6]. Once recurrence manifests after curative treatment, the prognosis is limited. Thus, to further improve patients’ chances of a survival, it is necessary to establish new approaches for treatment.

Recently, the preoperative nutritional status was shown to affect the occurrence of postoperative surgical complications, toxicity of adjuvant treatment, and patient’s survival in various malignancies [7–10]. Although several scoring system and serum biomarker have been tested, optimal nutritional biomarkers have not yet been established. If physicians could assess optimal serum nutritional biomarker, they might be able to make or change treatment strategies according to those biomarkers.

Prealbumin, a negative acute-phase protein synthesized in the liver, recently became the focus of research as a serum biomarker for assessing the nutritional status [11]. In addition, prealbumin may serve as a more sensitive marker than albumin due to its shorter half-life (about 1.9 days) [12]. Several studies have also reported that prealbumin might be a prognostic factor in patients with lung cancer, esophageal cancer, and renal cell carcinoma [13–15]. However, few studies have assessed the prognostic value of prealbumin in gastric cancer patients who have received curative treatment [16, 17]. In addition, the mechanism underlying the effect of prealbumin on the oncological outcomes in gastric cancer remains unclear.

Therefore, we investigated whether or not the overall survival (OS) and recurrence-free survival (RFS) were affected by the prealbumin level and clarified the clinical course according to the prealbumin level in gastric cancer patients who underwent curative treatment.

## Patients and Methods

### Patients

Patients were selected based on the medical records of consecutive patients who underwent curative resection for gastric cancer at Kanagawa Cancer Center from 2013 to 2017. The inclusion criteria were as follows: (1) histologically proven adenocarcinoma, (2) clinical stage I to III disease as evaluated using according to the 15th edition of the general rules for gastric cancer published by the Japanese Gastric Cancer Association [18], and (3) complete (R0) resection of gastric cancer with radical lymph node dissection.

### Surgical Procedure and Adjuvant Treatment

All of the patients received distal or total gastrectomy with lymphadenectomy. D1 + nodal dissection was performed for clinical stage IA disease, while D2 dissection was performed for clinical stage ≥ IB. Patients diagnosed with pathological II or III disease received adjuvant chemotherapy for one year. In principle, patients with pathological stage II disease received S-1 monotherapy, while those with pathological stage III disease received S-1 plus docetaxel or capecitabine + oxaliplatin therapy.

### Follow-up

Hematological tests and physical examinations were performed at least every 3 months for five years. The carcinoembryonic antigen and CA19-9 tumor marker levels were checked at least every 3 months for 5 years. Patients underwent computed tomography (CT) every 6–12 months until 5 years after surgery.

### Evaluations and Statistical Analyses

The significance of differences between the prealbumin levels and clinic pathological parameters was determined using the *χ*^2^ test. The Kaplan–Meier method was used to calculate the OS and RFS curves. Univariate and multivariate survival analyses were performed using a Cox proportional hazards model. *P* values of < 0.05 were considered to indicate statistical significance. The SPSS software program (v27.0 J Win; SPSS, Chicago, IL, USA) was used for all statistical analyses. This study was approved by the IRB of Kanagawa Cancer Center.

## Results

### Patients

We evaluated 447 patients in the present study. The median age was 68 (range: 32–90) years old, and 296 patients were male, while 151 were female. Based on the 3- and 5-year OS rate and previous studies, we set the cutoff value for prealbumin at 20 mg/dl in the present study. When comparing the background characteristics between patients with prealbumin < 20 mg/dl (low-prealbumin group) and prealbumin ≥ 20 mg/dl (high-prealbumin group), there were significant differences in the gender and clinical T and N status. The incidence rates of female patients and aggressive tumors were much higher in the low-prealbumin group than in the high-prealbumin group (Table 1).

**Table 1:** Patient characteristics

Characteristics	No. of patients (*n* = 447, %)	Low prealbumin group (*n* = 78)	High prealbumin group (*n* = 369)	*p* Value
Age (years)				0.075
< 65	135 (30.2%)	17 (21.8%)	118 (32.0%)	
≥ 65	312 (69.8%)	61 (88.2%)	251 (68.0%)	
Gender				< 0.001
Man	296 (66.2%)	29 (37.2%)	267 (72.4%)	
Woman	151 (33.8%)	49 (62.8%)	102 (27.6%)	
Pathological type				0.656
Intestinal	228 (51.0%)	38 (48.7%)	190 (51.5%)	
Diffuse	219 (49.0%)	40 (51.3%)	179 (48.5%)	
UICC T status				< 0.001
T1	276 (61.7%)	33 (42.3%)	190 (51.5%)	
T2 to T4	171 (38.3%)	45 (57.7%)	126 (48.5%)	
Lymph node metastasis				< 0.001
Negative	319 (71.4%)	43 (55.1%)	276 (74.8%)	
Positive	128 (28.6%)	35 (44.9%)	93 (25.2%)	
Lymphatic invasion				0.024
Negative	141 (31.5%)	33 (42.3%)	108 (29.3%)	
Positive	306 (68.5%)	45 (57.7%)	261 (70.7%)	
Vascular invasion				0.009
Negative	193 (43.2%)	44 (56.4%)	149 (40.4%)	
Positive	254 (56.8%)	34 (43.6%)	220 (59.6%)	
Postoperative complications				0.221
Yes	66 (14.8%)	15 (19.2%)	51 (13.8%)	
No	381 (85.2%)	63 (80.8%)	318 (86.2%)	

*UICC* Union for International Cancer Control

### Survival Analyses and Recurrence Patterns

**Fig. 1:**
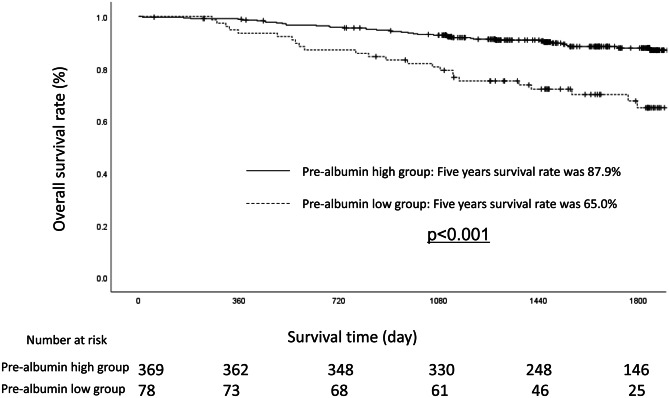
A comparison of the overall survival in the patients with a prealbumin level of ≥ 20 versus < 20 mg/dl

Each clinicopathological factor was categorized as shown in Table 2 and analyzed for its prognostic significance. Univariate analyses for the OS showed that the pathological T factor, pathological N factor, histological type, lymphatic invasion, vascular invasion, and prealbumin level were significant prognostic factors. The prealbumin level was therefore selected for the final multivariate analysis model. The OS rates at 3 and 5 years after surgery were 80.7% and 65.0% in the low-prealbumin group, respectively, and 93.1% and 87.9% in the high-prealbumin group, respectively, a statistically significant difference (*p* < 0.001). The OS curves are shown in Fig. 1.

**Table 2:** Uni- and multivariate Cox proportional hazards analysis of clinicopathological factors for overall survival

Factors	No	Univariate analysis			Multivariate analysis		
		OR	95% CI	*p* Value	OR	95% CI	*p* Value
Age (years)				0.129			
< 65	135	1.000					
65≦-	312	1.561	0.879–2.774				
Gender				0.209			0.098
Woman	151	1.000			1.000		
Man	296	1.414	0.824–2.427		1.643	0.913–2.955	
Pathological type				0.049			
Intestinal	228	1.000					
Diffuse	219	1.635	1.003–2.666				
UICC T status				< 0.001			0.073
T1	228	1.000			1.000		
T2–T4	219	4.518	2.675–7.632		1.816	0.946–3.487	
Lymph node metastasis				< 0.001			< 0.001
Negative	319	1.000			1.000		
Positive	128	5.203	3.166–8.550		2.939	1.618–5.337	
Pre-albumin level				< 0.001			0.002
20 < -	369	1.000			1.000		
-< 20	78	3.045	1.839–5.041		2.375	1.362–4.144	
Lymphatic invasion				< 0.001			
Negative	141	1.000					
Positive	306	3.072	1.896–4.978				
Vascular invasion				< 0.001			0.083
Negative	193	1.000			1.000		
Positive	254	4.197	2.443–7.210		1.736	0.930–3.238	
Postoperative complications				0.843			
Yes	66	1.000					
No	381	1.074	0.532–2.167				

*UICC* Union for International Cancer Control

**Fig. 2:**
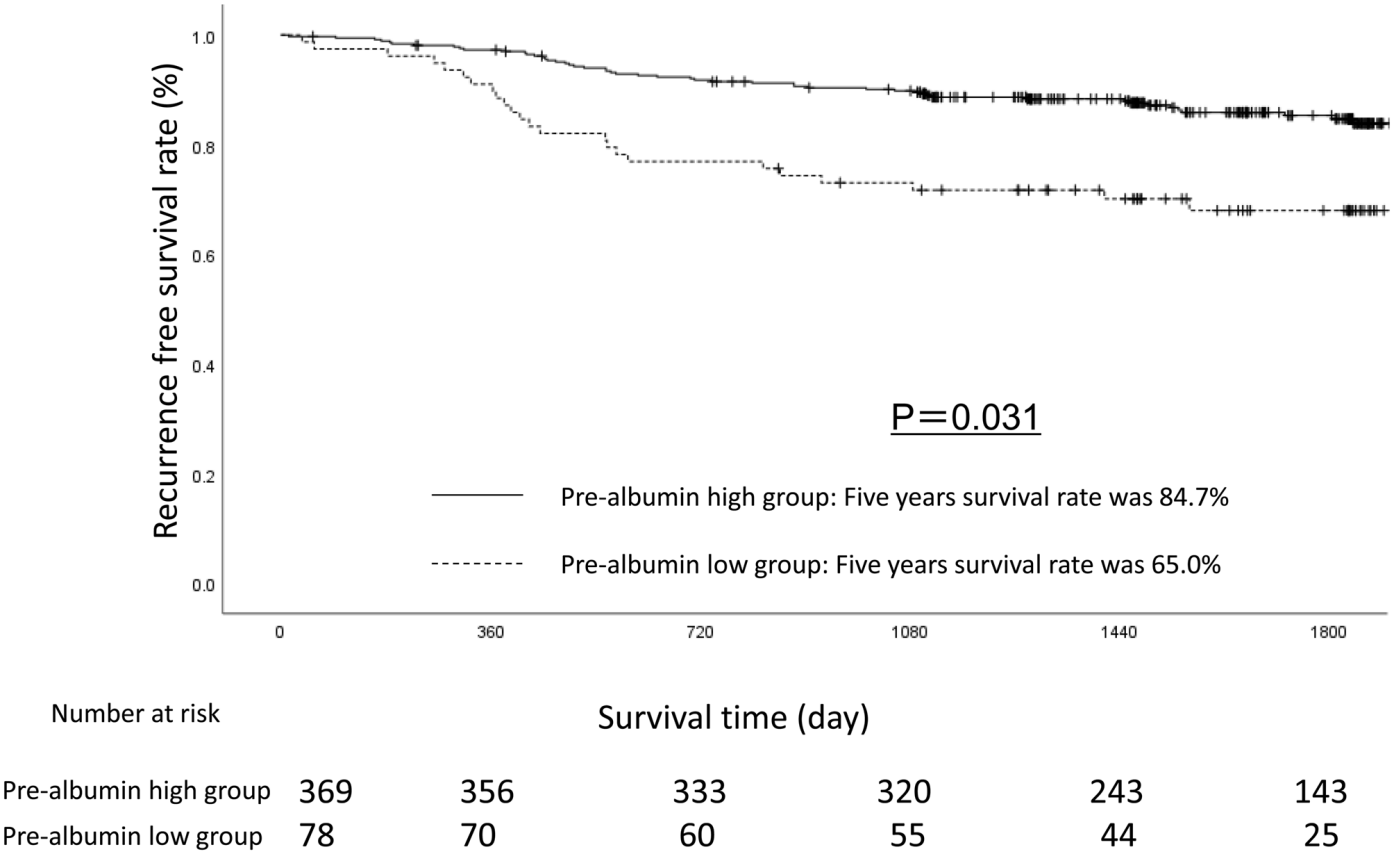
A comparison of the recurrence-free survival in the patients with a prealbumin level of ≥ 20 versus < 20 mg/dl

Univariate analyses for the RFS showed that the prealbumin level was a significant prognostic factor. It was thus selected as a significant prognostic factor for the final multivariate analysis model (Table 3). The RFS rates at 3 and 5 years after surgery were 71.7% and 68.0% in the low-prealbumin group, respectively, and 90.1% and 84.7% in the high-prealbumin group, respectively, a statistically significant difference (*p* = 0.031). The RFS curves are shown in Fig. 2.

**Table 3:** Uni- and multivariate Cox proportional hazards analysis of clinicopathological factors for recurrence-free survival

Factors	No	Univariate analysis			Multivariate analysis		
		OR	95% CI	*p* Value	OR	95% CI	*p* Value
Age (years)				0.140			
≤ 65	135	1.000					
65≦-	312	1.487	0.878–2.518				
Gender				0.366			
Woman	151	1.000					
Man	296	1.252	0.769–2.036				
Pathological type				0.077			
Intestinal	228	1.000					
Diffuse	219	1.500	0.957–2.351				
UICC T status				< 0.001			0.010
T1	228	1.000			1.000		
T2–T4	219	3.930	2.433–6.348		2.096	1.198–3.668	
Lymph node metastasis				< 0.001			< 0.001
Negative	319	1.000			1.000		
Positive	128	4.612	2.933–7.252		3.087	1.806–5.278	
Pre-albumin level				< 0.001			0.031
20 < -	369	1.000			1.000		
- < 20	78	2.488	1.553–4.036		1.724	1.050–2.832	
Lymphatic invasion				< 0.001			
Negative	141	1.000					
Positive	306	3.007	1.925–4.697				
Vascular invasion				< 0.001			
Negative	193	1.000					
Positive	254	3.470	2.144–5.617				
Postoperative complications				0.992			
Yes	66	1.000					
No	381	1.003	0.530–1.899

### Postoperative Adjuvant Chemotherapy Course and Recurrence Patterns in the High- and Low-Prealbumin Groups

When comparing the postoperative course between the high- and low-prealbumin groups, there were some differences in the postoperative adjuvant chemotherapy course. In the present study, 28.0% (125/447) of patients needed postoperative adjuvant chemotherapy. Among them, 48.7% (38/78) needed postoperative adjuvant chemotherapy in the low-prealbumin group, while 23.6% (87/369) needed postoperative adjuvant chemotherapy in the high-prealbumin group, a significant difference (*p* < 0.001).

The introduction rate of postoperative adjuvant chemotherapy differed between the two groups. Among the patients who required postoperative adjuvant chemotherapy, only 71.1% (27/38) received it in the low-prealbumin group, while 86.2% (75/87) received it in the high-prealbumin group, a significant difference (*p* < 0.001). The site of the first relapse differed significantly between the high- and low-prealbumin groups (Table 4). The incidence of peritoneal recurrence was significantly higher and lymph node metastasis marginally significantly higher in the low-prealbumin group than in the high-prealbumin group.

**Table 4:** Patterns of recurrence between the patients with pre-albumin level ≤ 20 and those with prealbumin level 20 ≤ -

			Prealbumin level				
	All cases (*n* = 447)		≤ 20 (*n* = 78)		20 ≤ - (*n* = 369)		
Recurrence site	Number	%	Number	%	Number	%	*p* Value
Peritoneal	22	4.9%	8	10.3%	14	3.8%	0.017
Hematological	15	3.4%	4	5.1%	11	3.0%	0.339
Lymph node	10	2.2%	4	5.1%	6	1.6%	0.057
Local site	8	1.8%	3	3.8%	5	1.4%	0.132
Total	45		19		26		

## Discussion

We evaluated whether or not the preoperative prealbumin level has any clinical significance with regard to the oncological outcomes of gastric cancer patients who receive curative treatment. We found that the patients with a prealbumin level < 20 mg/dl (low-prealbumin group) had significantly poorer outcomes than those with higher prealbumin levels (high-prealbumin group). In addition, the incidence of the introduction of postoperative adjuvant chemotherapy was significantly lower in the low-prealbumin group than in the high-prealbumin group. Therefore, our results suggest that the preoperative prealbumin level is a promising clinical prognostic marker for gastric cancer patients who receive multimodal treatment, and patients with a low prealbumin level require close attention in their postoperative clinical course, especially with regard to adjuvant chemotherapy.

We found that the preoperative prealbumin level was a viable prognostic factor for gastric cancer patients. The hazard ratio (HR) for the OS was 2.375 (95% confidence interval [CI]: 1.362–4.144), and that for RFS, it was 1.724 (95% CI: 1.052–2.832). Similar HRs were observed in previous studies. Han et al. evaluated the prognostic impact of preoperative prealbumin in 101 adenocarcinomas of esophagogastric junction (AEG) patients [16]. They set the cut-off value of prealbumin at 200 g/l and found that the prealbumin level was indeed a prognostic factor, with a high prealbumin level (≥ 200 g/l) associated the longer OS in AEG patients than a low level. The HR for the OS (prealbumin < 200 g/l for prealbumin > 200 g/l) was 0.494 (95% CI: 0.271–0.901, *p* = 0.021). In addition, Shen et al. evaluated the clinical significance of preoperative prealbumin in 731 stage II and III gastric cancer patients [17] with a cut-off value of 180 mg/l. They found that the preoperative prealbumin level was also a prognostic factor in their study. The median OS was 62 months in the high-prealbumin group and 46 months in the low-prealbumin group (HR: 1.362, 95% CI: 1.094–1.695), and the median RFS was 61 months in the high-prealbumin group and 37 months in the low-prealbumin group (HR: 1.369, 95% CI: 1.099–1.706). In both the present and previous studies, a high level of prealbumin demonstrated a positive association with the survival in gastric cancer patients.

However, there remain some concerns associated with the use of the preoperative prealbumin level as a prognostic factor for gastric cancer. First, the optimal cutoff value and method of evaluating the prealbumin level are unclear. In the present study, we set the cutoff value according to the 3- and 5-year survival rates. Han et al. and Shen et al. set their cutoff values using a receiver operating characteristic (ROC) curve analysis based on the most prominent points on the ROC curves [16, 17]. While the cutoff values have been similar, standard evaluation methods need to be established. Second, the evaluation point of prealbumin is also important. In the present study, we measured the prealbumin level within three to four days before surgery, while the other two studies measured the level within 7 days before surgery. The half-life of prealbumin is about 1.9 days [11, 12]. Thus, the timing of the evaluation of prealbumin might affect the cutoff value. A further analysis is needed to confirm the utility of prealbumin as a prognostic factor in daily clinical practice.

We also found that the prealbumin level influenced the introduction of postoperative adjuvant chemotherapy and recurrence pattern. In the low-prealbumin group, almost half of the patients needed postoperative adjuvant chemotherapy, with 70% of them actually receiving adjuvant chemotherapy. In contrast, in the high-prealbumin group, only 20% of the patients needed postoperative adjuvant chemotherapy, with nearly 90% of them actually receiving adjuvant chemotherapy. Therefore, the merits of adjuvant chemotherapy were limited in the low-prealbumin group. In addition, the difference in the rate of the introduction of adjuvant chemotherapy affected the recurrence pattern. Peritoneal recurrence was significantly more frequent, and lymph node metastasis was marginally more frequent in the low-prealbumin group than in the high-prealbumin group. Several previous pivotal studies showed similar results. For example, the ACTS-GC trial, which assessed the usefulness and efficacy of S-1 adjuvant chemotherapy for locally advanced gastric cancer, demonstrated that effective adjuvant chemotherapy significantly suppressed and reduced peritoneal and lymph node recurrence [19]. The recurrence rates at the peritoneum and lymph node were 11.2% and 5.1%, respectively, in the adjuvant treatment group and 15.8% and 8.7%, respectively, in the surgery alone group in the ACTS-GC trial (*p* = 0.009 and *p* = 0.01, respectively). Given these previous findings, the prealbumin level was considered to have affected the rate of introduction of adjuvant chemotherapy, and this decreased rate of chemotherapy introduction affected the pattern of recurrence and resulted in a poor prognosis.

Several limitations associated with the present study warrant mention. First, the present study was retrospective in nature and conducted at a single center, and the sample size was relatively small. Second, there was some degree of selection bias. In our institution, the surgical indication was determined by seven physicians, including an anesthesiologist, who took into consideration the activities of daily living, performance status, medical history, physical examination findings, and organ function, as is done in general community hospitals. However, there is a possibility that only patients with a good status were selected, as our hospital is a regional cancer center that treats only cancer patients. Elderly patients with co-morbidities who visit general hospitals often undergo surgery at the hospital at which they were diagnosed with gastric cancer. Indeed, the ASA physical status, incidence of co-morbidities, and preoperative laboratory values were lower than in general hospital patients. Therefore, the gastric cancer patients in the present study were well selected and fit for surgery. Considering these limitations, the findings of our study should be validated in another cohort.

In conclusion, prealbumin was determined to be a useful risk factor for the survival in patients who undergo curative treatment for gastric cancer. It is necessary to develop an effective plan of perioperative care and perioperative treatment strategy according to the prealbumin level.
